# Editorial: Materialities of Age and Ageing

**DOI:** 10.3389/fsoc.2019.00014

**Published:** 2019-03-04

**Authors:** Grit Höppner, Monika Urban

**Affiliations:** ^1^Catholic University of Applied Sciences of North Rhine–Westphalia, Münster, Germany; ^2^Department 11 Human and Health Sciences, University of Bremen, Bremen, Germany

**Keywords:** material gerontology, Gerontological research, materialities of age and ageing, non-human, Editorial

Since the end of the 1990s the so-called material turn in the social sciences, cultural studies, and the humanities has made it clear that not only people create and reproduce the social world, but so-called “nonhuman” actors are also involved in these processes (e.g., Haraway, 1991; Barad, 2003; Latour, 2005). The fact that these various materiality-related theoretical and conceptual approaches have so far found their way into the gerontological debate only at isolated intervals (e.g., Twigg, 2013; Gilleard and Higgs, 2015) is astonishing. The aim of the research topic, “Materialities of Age and Ageing,” is to explore what we learn if we look at age and ageing from the perspective of materiality-related theories and concepts, such as material sociology, material culture studies, science and technology studies, and new materialism. In our understanding, “Materialities of Age and Ageing” comprises both the fleshy-sensual experiences of human bodies and their interplay with and relation to non-humans, such as commodity items, things, technologies, architecture, and spaces. The authors of the research topic will not essentialize human and non-human kinds of materiality as natural facts.

The research topic contributes to a new material gerontology. In their introductory contribution the two guest editors, Grit Höppner and Monika Urban, propose the term material gerontology. This term was further developed by a network of scholars working in the fields of sociology, cultural gerontology, science and technology, and gender studies (https://materialgerontology.wordpress.com/veranstaltungen-des-netzwerks/). They foster a debate on roles, meanings, and functions of non-humans in the production of age and ageing—and some of these scholars have contributed to the research topic at hand. What the authors of the research topic and the scholars of the network have in common is both their skepticism toward the idea that age and ageing take place solely in humans and their openness to considering non-human kinds of materiality as being involved in ageing processes. As a starting point, all authors provide information on how the theoretical foundations of cultural gerontology and critical gerontology can be explored from a theoretical perspective on materiality. The aim is not only to enrich the understanding of material gerontology but also to relieve human subjects of some of the burden of an inexorable ageing process; thus, it relativizes the responsibility ascribed to seniors to perform a “successful” and therewith delayed ageing process.

“Materialities of Age and Ageing” includes four papers that theorize age and ageing from a materialist perspective, two papers that add empirical insides to a material gerontology, and one book review.

In an introductory contribution the two guest editors, Höppner and Urban, outline a material understanding of ageing processes. In “Where and How Do Ageing Processes take place in Everyday Life? Answers from a New Materialist Perspective,” they review social constructivist ideas and cultural and critical gerontological assumptions on conceptualizations of bodies and agencies of ageing and rethink them using new materialist concepts, Höppner and Urban point out the importance of both the material-discursive co-production of ageing processes and the decentralization of the human actor.

Wanka and Gallistl turn to the influence of digital technology in postmodern societies and digital infrastructures, which are substantially integrated into the everyday lives of older people. In “Doing Age in a Digitized World—A Material Praxeology of Ageing with Technology” the authors attempt to both “praxeologize” and “materialize” current studies of ageing and technologies by introducing the theoretical model of “material praxeology of ageing with technology.” Wanka and Gallistl give an example of the application of this model in a research project in the field of Active and Assistive Living that took place in Vienna, Austria.

Müller scrutinizes the status of care work in capitalist societies in “The careless society—dependency and care work in capitalist societies.” The problematisation of actual lack of sensual and emotional relationships in the context of professional care work takes Müller to develop an care-ethical position. Making reference to feminist phenomenology, Müller presents her concept of “value abjection.”

“Doing Age and Doing Desire in and Through Film. Queer Perspectives on Gender, Ageing, and Desire,” addresses the intersections of “doing age” and “doing desire” in films. Analyzing five films, Eckert and Martin develop a taxonomy of the various forms of desire displayed in them. Their central hypothesis is that the films do not just represent desire in old age but—in and through the desire that they produce in the film—they also materialize desire in the spectators. They conclude that films allow for a specific corporeal-somatic experience beyond a simple and normalized heterosexuality in old age.

Bergschöld deals with training programs for nursing students learning how to care for elderly patients with dementia. “Configuring Dementia; How Nursing Students Are Taught to Shape the Sociopolitical Role of Gerontechnologies” draws on ethnographical fieldwork to investigate lectures in which nursing students learn about technologies in dementia care. Bergschöld concludes that the way the students are currently taught to deal with the selection and placement of the technologies necessarily contributes to a disempowerment of older adults with dementia who are ageing at home.

In “Materialities in and of Institutional Care for Elderly People” Artner explores a parallel between nursing homes and the “total institution” (Goffman). By drawing on Goffman's ideas on the creation and presentation of the self, she traces the effect produced by the placement and handling of material objects in the course of the performance of nursing work. By using empirical examples, she demonstrates how social interactions lead to the institutionalization of the residents.

A book review completes the research topic. Urban reviews the recently published anthology titled “Ageing as Social and Cultural Praxis. Orders—Relationships—Materiality” (Endter and Kienitz, 2017). This anthology, available only in German, considers ageing not as a biological process associated with deteriorating health, but rather as a social practice. It gives insights into how ageing is performed within a social and cultural order, as being interwoven within human and non-human relationships (e.g., things or architecture).

The research topic impressively shows that it is worth decentralizing the human actor that is usually focused on in research on age and ageing. This decentralization not only provides impulses for a new research on age and ageing but also enriches in general the social sciences that take human and non-human kinds of materiality into account.

## Author Contributions

The ideas that are presented in this editorial are developed by GH and MU. GH and MU wrote the first draft of the manuscript together, contributed to manuscript revision, and read and approved the submitted version.

### Conflict of Interest Statement

The authors declare that the research was conducted in the absence of any commercial or financial relationships that could be construed as a potential conflict of interest.
